# Supplementary material for: Splice-Junction-Based Mapping of Alternative Isoforms in the Human Proteome
Source: Cell Rep. Author manuscript; Available in PMC 2020 Jan 15. (PMC6961840; doi:10.1016/j.celrep.2019.11.026)
Supplement: 2 [file NIHMS1546469-supplement-2.zip › DF1/PXD010154/Heart/Heart_86_AKT1_MLPTYPHSR.pdf]

A

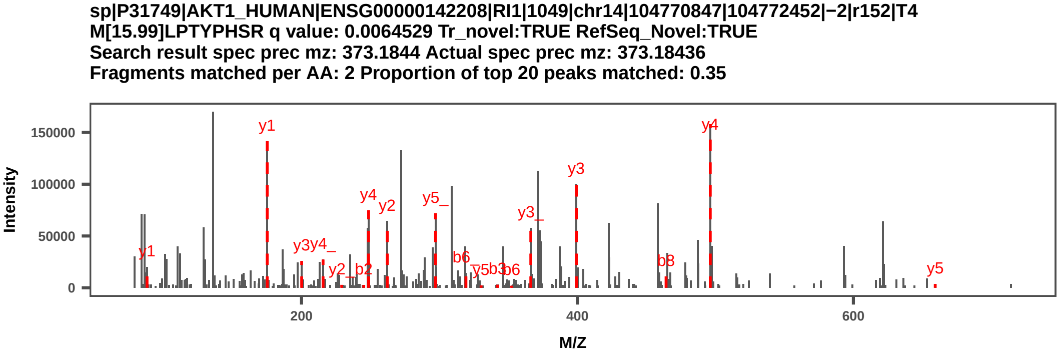
sp|P31749|AKT1_HUMAN|ENSG00000142208|RI1|1049|chr14|104770847|104772452|−2|r152|T4
M[15.99]LPTYPHSR q value: 0.0064529 Tr_novel:TRUE RefSeq_Novel:TRUE
Search result spec prec mz: 373.1844 Actual spec prec mz: 373.18436
Fragments matched per AA: 2 Proportion of top 20 peaks matched: 0.35
Intensity
150000
100000
50000
0
200
400
600
M/Z
y1
y1
y3y4_
y2_
b2
y4
y2
y5_
b6
y5b3
b6
y3_
y3
b8
y4
y5

B

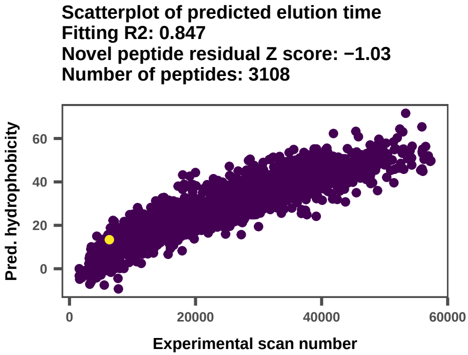
Scatterplot of predicted elution time
Fitting R2: 0.847
Novel peptide residual Z score: −1.03
Number of peptides: 3108
Pred. hydrophobicity
60
40
20
0
0
20000
40000
60000
Experimental scan number

C

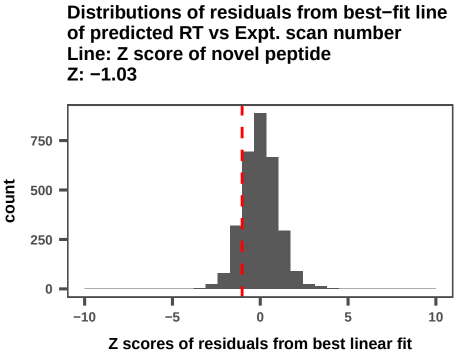
Distributions of residuals from best−fit line
of predicted RT vs Expt. scan number
Line: Z score of novel peptide
Z: −1.03
count
750
500
250
0
−10
−5
0
5
10
Z scores of residuals from best linear fit
